# Measuring Nanoscale Distances by Structured Illumination Microscopy and Image Cross-Correlation Spectroscopy (SIM-ICCS)

**DOI:** 10.3390/s21062010

**Published:** 2021-03-12

**Authors:** Isotta Cainero, Elena Cerutti, Mario Faretta, Gaetano Ivan Dellino, Pier Giuseppe Pelicci, Alberto Diaspro, Luca Lanzanò

**Affiliations:** 1 Nanoscopy and NIC@IIT, CHT Erzelli, Istituto Italiano di Tecnologia, Via Enrico Melen 83, Building B, 16152 Genoa, Italy; isotta.cainero@iit.it (I.C.); elena.cerutti@iit.it (E.C.); 2 DIFILAB, Department of Physics, University of Genoa, Via Dodecaneso 33, 16143 Genoa, Italy; 3 Department of Physics and Astronomy “Ettore Majorana”, University of Catania, Via S. Sofia 64, 95123 Catania, Italy; 4 Department of Experimental Oncology, IEO, European Institute of Oncology IRCCS, 20100 Milan, Italy; mario.faretta@ieo.it (M.F.); gaetano.dellino@ieo.it (G.I.D.); piergiuseppe.pelicci@ieo.it (P.G.P.); 5 Department of Oncology and Hemato-Oncology, University of Milan, 20100 Milan, Italy

**Keywords:** super-resolution microscopy, structured illumination microscopy, SIM, image correlation spectroscopy, image cross-correlation spectroscopy, ICCS, colocalization

## Abstract

Since the introduction of super-resolution microscopy, there has been growing interest in quantifying the nanoscale spatial distributions of fluorescent probes to better understand cellular processes and their interactions. One way to check if distributions are correlated or not is to perform colocalization analysis of multi-color acquisitions. Among all the possible methods available to study and quantify the colocalization between multicolor images, there is image cross-correlation spectroscopy (ICCS). The main advantage of ICCS, in comparison with other co-localization techniques, is that it does not require pre-segmentation of the sample into single objects. Here we show that the combination of structured illumination microscopy (SIM) with ICCS (SIM-ICCS) is a simple approach to quantify colocalization and measure nanoscale distances from multi-color SIM images. We validate the SIM-ICCS analysis on SIM images of optical nanorulers, DNA-origami-based model samples containing fluorophores of different colors at a distance of 80 nm. The SIM-ICCS analysis is compared with an object-based analysis performed on the same samples. Finally, we show that SIM-ICCS can be used to quantify the nanoscale spatial distribution of functional nuclear sites in fixed cells.

## 1. Introduction

In healthy cells, biological processes need to be precisely coordinated both in time and space [1]. An example is represented by DNA replication and transcription processes. In healthy eukaryotic cells, these two processes are spatio-temporarily segregated. In tumors they are frequently not coordinated and compete with each other for the same DNA double strand. This competition can cause the local physical collision of replication and transcription machineries causing DNA damage and genomic instability which can lead to cancer development [2,3,4,5,6].

In order to study the spatial distribution of molecules within single-cells, one of the most powerful techniques is fluorescence microscopy. The study of the spatial distribution of molecules can be performed by labeling different molecular species in multiple colors and observe them on different channels of the microscope [7]. The simplest way to quantify the relative distribution of two molecular species by optical microscopy is by analyzing whether there is an overlap of the signals from each channel. Indeed, the color-mixing gives information on the colocalization of the target molecules within the cell environment.

However, in optical microscopy, the spatial scale of colocalization is strictly linked to the resolution of the microscope. In conventional optical microscopy, the maximum achievable resolution is limited by diffraction to ~250 nm and, consequently, all the molecules separated by the distance of 250 nm or less will be overlapped and, thus, potentially considered as colocalized. To overcome this problem and get information at the nanoscale, multiple distance-sensing approaches have been developed. One example is Forster resonance energy transfer (FRET) [8,9] which is a precise technique to define molecular interactions thanks to the energy transfer occurring from an excited donor fluorophore to an acceptor fluorophore via dipole-dipole coupling, thus probing distances smaller than ~10 nm. A completely different technique is in situ proximity ligation assay (PLA) in which two antibodies are functionalized with oligonucleotides [10]. When these two antibodies link to target molecules, whose proximity is smaller than ~40 nm, the oligonucleotides will link and start the amplification creating a circular DNA molecule. This molecule is then recognized by functionalized probes, allowing fluorescence detection.

The introduction of super-resolution (SR) optical microscopy changed this scenario allowing us to overcome the diffraction limit with different techniques, reaching different nanoscale levels [11,12]. For instance, Single-molecule localization microscopy (SMLM) [13], achieves ~20 nm nanometer resolution by repeatedly switching ‘photo-switchable’ fluorophores on and off to precisely map their x, y, z position, to reconstruct the final super-resolution image. Another SR technique is stimulated emission depletion (STED) [14,15] microscopy in which the excitation laser beam excites the fluorophores within the point spread function (PSF) while a superimposed doughnut-shaped laser beam depletes the invested fluorophores located in the periphery of the PSF, through stimulated emission, producing a smaller effective PSF. This “shrinking” of the observation volume allows reaching ~40 nm resolution. Another super-resolution approach emerging as a tool to study the cellular environment is structured illumination microscopy (SIM). In this technique, the sample is excited with a shifting striped pattern to access the sub-diffraction features. This pattern is then oriented in different angles and, after multiple raw striped acquisitions, the super-resolution image is reconstructed and it can reach ~100 nm resolution [16]. Despite this modest increase in spatial resolution SIM is very popular because is a ‘gentle’ super-resolution technique [12] due to the low laser power used, because it can perform multicolor acquisitions using conventional antibodies and also because it can perform 3D sectioning.

All these techniques can be used to perform multi-color images and to study the distributions of target molecules. To quantify and analyze the value of colocalization in multi-color super-resolution images, there are mainly two approaches: object-based and pixel-based methods [17].

The *object-based approaches* foresee the segmentation of the sample into single objects with precise coordinates to analyze their distribution and their relative spatial organization. They can be used on any microscope data set as long as the molecules of interest are well resolved [18]. They are widely used in SMLM measurements because the acquired data is already a list of coordinates of each particle [19,20,21]. It can be used in all the other microscopy techniques, with a prior segmentation of the images into objects. Object-based methods provide a map of the whole sample features and allow performing statistical analysis of the relative distributions of the molecules of interest.

The *pixel-based methods* do not need pre-segmentation of the image. Instead, they rely on the extraction of colocalization or correlation coefficient from each pixel intensity of the reconstructed data [22,23]. For this reason, these methods can be used with every microscopy data set to quantify relative distributions in multicolor channel acquisitions.

Among multiple pixel-based approaches, image cross-correlation spectroscopy (ICCS) is an attractive method. ICCS was developed from fluorescence cross-correlation spectroscopy (FCCS) that is the two-color version of fluorescence correlation spectroscopy (FCS) [24]. With FCCS is possible to study dynamic interactions, namely the value of interacting particles is calculated by analyzing the intensity fluctuations in time caused by variation of fluorophore concentrations in the observation volume [25,26]. Indeed, it has been demonstrated that the same principle of FCS and FCCS can be applied to study spatial intensity fluctuation in images. In fact, ICS and ICCS correspond to the spatial versions of FCS and FCCS respectively.

Therefore, ICCS can be used to investigate the spatial distribution of particle in images as a pixel-based method [27,28]. ICCS has been recently combined with super-resolved STED microscopy [29] to analyze the nanoscale spatial distribution of functional sites in the cell nucleus. Oneto et al. have applied ICCS to dual-color STED images of nuclei in fixed cells. They have shown that with STED-ICCS it is possible to extract information of a colocalized fraction from the cross-correlation function. In addition, they have shown that it is possible to extract an average distance value of correlated particles in the sample, proving that STED-ICCS can be a valuable method to sense molecular distances within cells. It would be interesting to extend this type of analysis to multi-color SIM images. To the best of our knowledge, in the context of SIM, ICCS has been only coupled to TIRF-SIM measurements [30].

Here, we show that the combination of SIM and ICCS (SIM-ICCS) can be used to perform colocalization analysis and measure nanoscale distances between two molecular species. We validate the method on SIM images of a model sample, which is based on DNA origami fabrication. In this sample, fluorophores of two different colors are located at the specific distance of 80 nm. We perform SIM-ICCS and compare the results with an object-based analysis performed on the same SIM images. The results show that SIM-ICCS is a powerful tool to measure nanoscale distances. As an application, we show that SIM-ICCS can be used to quantify the colocalization of functional nuclear sites in fixed cells.

## 2. Materials and Methods

### 2.1. Samples

A sample of GATTA-SIM Nanoruler 160RBR was purchased from Gattaquant DNA Nanotechnologies (Munich, Germany) and used to perform two-color distance analysis. Every single nanoruler is composed by three fluorophores, ATTO 647N—Alexa Fluor^®^ 488—ATTO 647N (Gattaquant DNA Nanotechnologies, Munich Germany), spaced 80 nm apart from each other. U937 cells (U937-PR9 subclone) were grown in Roswell Park Memorial Institute medium (RPMI-1640 medium from Sigma Aldrich, St. Louis, MO, USA) supplemented with 10% fetal bovine serum (ECS0180L, Euroclone, Pero, Italy) and 1% penicillin/streptomycin (Sigma Aldrich). In particular, cells were grown in their flasks and then transferred to 14 mm glass coverslips 1.5 high precision for imaging and fixed with 4% paraformaldehyde (*w/v*), PFA, at RT for 10 min.

### 2.2. Immunostaining

The positive control was stained with 5-ethynyl-2′-deoxyuridine, EdU, and proliferating cell nuclear antigen (PCNA). EdU was used to stain replication sites of nascent DNA and the staining was performed by using a Click-iT reaction by Invitrogen™ (Carlsbad, CA, USA). EdU was incubated, 10 µM, in the cell’s flask for 25 min at 37 °C, then cells were plated and fixed with a 10 min incubation of 4% paraformaldehyde (*w/v*), PFA at RT. Next, cells were washed twice with 3% BSA and then permeabilized with 0.5% Triton 100X for 20 min. EdU detection was performed with the Click-iT EdU imaging kit by Thermo-Fisher Scientific (Waltham, MA, USA), using the Alexa azide 488. The PCNA staining was performed, after 1-h blocking buffer (BB) incubation at RT, by using a dilution [1:2000] of primary antibody Rat anti-PCNA by Chromotek (16D10, Planegg, Germany) in the BB overnight at 4 °C. The BB solution consists of 3% Sigma Aldrich bovine serum albumin (BSA) and 0.5% Triton 100X. The next morning the sample was washed once in BB solution and twice in the washing buffer (WB) solution, which is made by 0.2% BSA and 0.06% Triton 100X. The cells were then incubated with the secondary antibody for 1-h, which was anti-rat ATTO 594 [1:500] dilution in BB, then they were washed once in BB, twice in WB and three times in phosphate buffered saline (PBS) solution.

The negative control was stained with histone H3K9me2, and RNA polymerases, RNApol2ser2P. Both these stainings were performed after 1-h BB solution incubation at RT. The RNA polymerases RNApol2ser2P, which is associated with productive transcriptional elongation, and H3K9me2, which marks silenced chromatin, were stained with an 4 °C overnight incubation with an Abcam (Cambridge, UK) rabbit anti-RNApol2ser2P (ab5095), in a [1:500] dilution, and Abcam mouse anti-H3K9me2 (ab1220). Then cells were washed once in BB and twice in WB. Next, cells were incubated for 1-h at RT with the secondary antibodies anti-rabbit ATTO 594 in BB [1:500] and anti-mouse Alexa 488, [1:1000] dilution. Then, samples were washed once in BB, twice in WB and thrice in PBS.

All the samples prepared for this work were also incubated 20 min with Hoechst (33342 Thermo Fisher Scientific, Waltham, MA, USA) as a counterstain for the DNA and mounted overnight by using the Invitrogen ProLong Diamond Antifade Mounting Medium (P36965).

### 2.3. Experiments

All the acquisitions were performed using a N-SIM Super-Resolution Microscope equipped with a 1.49 NA 100× objective (CFI Apo TIRF 100xc Oil, Nikon, Tokyo, Japan) and with the 3D EX V-R 100×/1.49 Grating Block. The laser excitation was provided by a 4-laser unit equipped with 405, 488, 561 and 640 nm. Hoechst was excited at 405 nm, and was detected from 440 to 485 nm. Alexa 488 excitation was performed at 488 nm and the emission was collected between 515–545 nm. ATTO 594 was excited at 561 nm and the emission was detected at 590–640 nm. ATTO 647N was excited at 640 nm and its emission collected between 665 nm and 738 nm due to the filters mounted on our microscope.

### 2.4. Image Cross Correlation Spectroscopy (ICCS) Analysis

Oneto et al. [29] provided an open-source code that was used in this work to perform Image correlation spectroscopy (ICS) and Image cross-correlation spectroscopy (ICCS) in MatLab (The Mathworks, Natick, MA, USA). The analysis is based on the calculation of spatial autocorrelation (ACF) and cross-correlation (CCF) functions. In particular, 2D image correlation functions were calculated as:

G_ij_ (δ_x_, δ_y_) = <I_i_(x,y)I_j_(x + δ_x_, y + δ_y_)>/<I_i_(x,y)> <Ij(xy)> (1)

In Equation (1), the angles in brackets indicate the average over all the pixels from the image, and I_1_(x,y) and I_2_(x,y) correspond to the images in the two-channels. To calculate the ACF we set i = j = 1 and i = j = 2. To calculate the CCF, we set i = 1 and j = 2. The numerator in the equation, I_i_(x,y)I_j_(x + δ_x_, y + δ_y_), is calculated with a fast 2D Fourier transform algorithm. Next, the 2D correlation functions were converted into one-dimensional radial correlation functions, G_ij_ (δ_r_) by performing an angular mean as explained in Scipioni et al. [31]. Then, a Gaussian fit of the calculated radial 1D correlation functions is performed to find the values of the amplitude, G_ij_ (0), and the width parameters, w_ij_^2^, as follows:

G_ij_ (δ_r_) = G_∞_ + G_ij_ (0) exp(−δ_r_^2/^w_ij_^2^) (2)

The width parameter corresponds to the 1/e^2^ of a Gaussian function and it is related to the full width half maximum (FWHM) by the relationship w = FWHM/(2ln2)^1/2^. To calculate the co-localization coefficients M_1_ and M_2_ we used the amplitude parameter M_1_ = G_12_ (0)/G_22_ (0) and M_2_ = G_12_ (0)/G_11_ (0). To calculate the co-localized fraction *f_ICCS_* we performed the arithmetic average of M_1_ and M_2_ coefficients.

The broadening of the cross-correlation function with respect to the autocorrelation functions was evaluated as Δw = w_12_-w_cc_ with w_cc_ = ((w_11_^2^ + w_22_^2^)/2)^1/2^, and the Δw value was used to calculate a distance value d_ICCS_ = (Δw/c)^1/2^, where c is a factor determined from simulated data, as explained in Oneto et al. [29].

### 2.5. Object-Based Analysis

The object-based analysis was performed following the one presented in Oneto et al., by using an ImageJ plugin called JACoP [17]. This plugin requires, as first step, a manual setting of the threshold to select the particles to be identified in each channel. The second step requires setting the pixel size of the image, the wavelength used, the Numerical Aperture of the objective, and the refractive index. The minimum particle size was set at 4 pixels, considering that our pixel size was 33 nm and our resolution was ~135 nm. The plugin provides a set of data comprehending x, y coordinates for each particle in each channel and the value of distances between each molecule of the first channel from all the particles of the second channel. We used these values of distances to build the relative distance distribution (RDD) histogram. To analyze the colocalization peak we subtracted the random component of the histogram, which was calculated through a linear fit forced through the origin, in a distance range from 250 nm to 500 nm. The calculated random component was then subtracted from the cumulative data, obtaining ‘only’ the colocalization peak. We performed a Gaussian fit of the colocalization peak in order to extract the mean distance value.

## 3. Results

### 3.1. SIM Imaging of Optical Nanorulers

**Figure 1:**
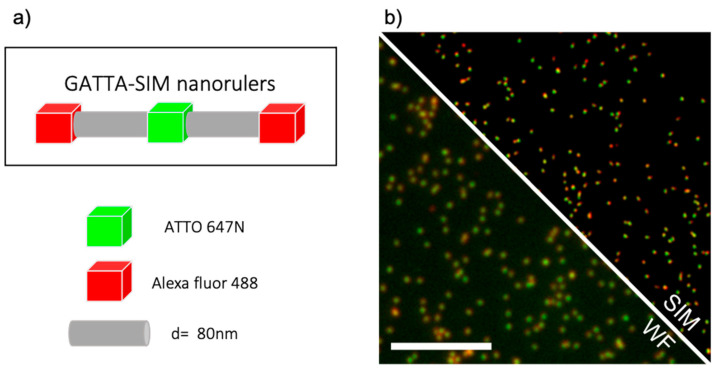
Schematic of the optical nanoruler and SIM imaging. (**a**) Schematic representation of GATTA-SIM nanorulers. Each nanoruler presents two groups of red fluorophores at both ends, ATTO 647N, and one central group of Alexa fluor 488 green fluorophores spaced from the two red ones by 80 nm. (**b**) Comparison between Widefield and N-SIM reconstructed image of GATTA-SIM nanorulers, ROI 256 × 256 pixels, scalebar 5µm.

This work aims to compare the use of an object-based method and super-resolved ICCS to measure nanoscale distances in two-color SIM images. To demonstrate that super-resolved ICCS can extract correlation distances, we performed both analyses on a model sample, optical nanorulers. These are DNA-origami-based structures in which two colors are located at a precise distance, as shown in Figure 1. Each nanoruler is equipped at both ends with a group of red fluorescent molecules, ATTO 647N, spaced by 160 nm, and with a central group of green fluorophores, Alexa 488, outdistanced from both red fluorophores by 80 nm distance.

**Figure 2:**
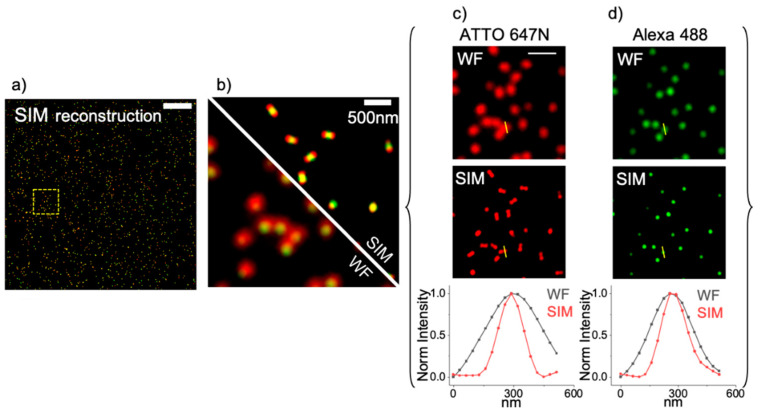
Evaluation of the optical resolution achieved by SIM imaging. (**a**) 1024 × 1024 pixel SIM reconstructed Image, scale bar 5 µm. (**b**) 128 × 128 pixel ROI from image (**a**). Shown are Widefield acquisition, bottom left, and SIM acquisition top right, scalebar 500 nm. (**c**) Analysis on the red channel: shown are the Widefield image (top), the SIM reconstruction (middle) and the intensity profiles corresponding to the yellow lines (bottom). The values of resolution extracted from these profiles are r_WFred_ = 350 nm; r_SIMred_ = 137 nm. (**d**) Analysis on the green channel: shown are the Widefield image (top), the SIM reconstruction (middle) and the intensity profiles corresponding to the yellow lines (bottom). The values of resolution extracted from these profiles are r_WFgreen_ = 260 nm; r_SIMgreen_ = 150 nm.

At first, we checked and characterized the resolution achieved in these samples by our microscope, as shown in Figure 2, by performing a line profile analysis. In this analysis, we plot the fluorescence intensity vs. the nanometer distances, and perform a Gaussian fit from which we extract the value of the FWHM. Eventually, we calculated the mean value of *n* = 15 particles for both the green and the red channel. In the end, we estimate ~140 nm resolution in the green channel, Alexa 488, and ~135 nm resolution in the red channel, ATTO 647N. Reaching 135 nm in the red channel allowed separating most of the nanorulers imaged in the red channel, whilst in the widefield acquisition, they are not resolved. This improvement in resolution allows us to analyze relative distances in the SIM acquisitions of nanorulers and to accurately measure the distances between the red and the green channels.

### 3.2. Object-Based Analysis of SIM Images of Optical Nanorulers

**Figure 3:**
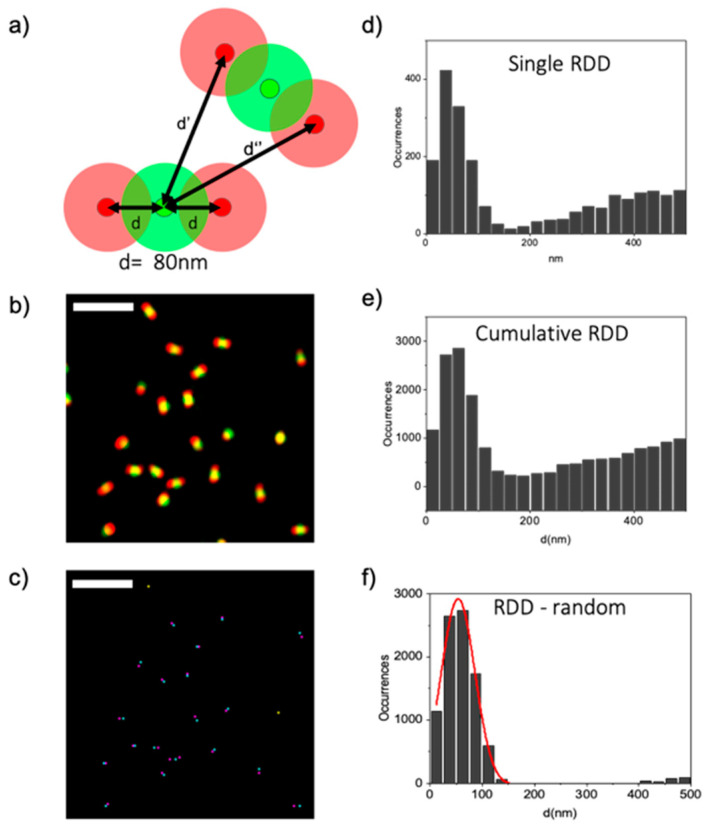
Object-Based analysis of optical nanorulers. (**a**) Schematic representation of the extraction of distances from the object-based analysis. Shaded red and green circles represent the SIM PSF in the two channels. (**b**) 128 × 128 pixels ROI of SIM reconstructed super-resolution image. (**c**) Map of the centers of mass of the identified objects. Magenta and Cyan spots stands for molecules in the red and green channel respectively, whose distance is 30 nm < d <160 nm, whilst the yellow spots stand for structure whose center of mass is closer than 30 nm, which corresponds to our pixel size. (**d**) Relative distance distribution (RDD) histogram calculated from a single 1024 × 1024 SIM image. (**e**) Cumulative RDD histogram of *n* = 11 samples considered in the analysis. (**f**) Gaussian fit of the RDD histogram without the random component. The analysis of the co-localization peak in all the eleven samples gave a mean distance of d = 54 ± 32 nm (Mean ± SD).

After testing the resolution of the acquired SIM images, we characterized the sample through an object-based method and we analyzed *n* = 11 SIM images of nanorulers. We chose the ImageJ plugin JACoP to perform the object analysis from which we get a list of coordinates of each center of mass detected in the image and a map of the center of mass, as shown in Figure 3c. For each sample, we calculated a histogram of the values of distance between red and green particles, which we call a relative distance distribution (RDD) histogram. In the case of non-colocalizing samples, i.e., red and green particles distributed randomly in relation to each other, we expect to obtain a linear growing component due to simple geometrical considerations (i.e., at distance *d* from a given particle, there is a number of particles proportional to 2π*d*) [29]. In the case of nanorulers, we obtain a peak of colocalization superimposed to a linear component (Figure 3d). The colocalization peak corresponds to distances between particles on the same nanorulers, the random component corresponds to distances between particles on different nanorulers. Then, we gather the data from all the images and we build the cumulative distribution graph in Figure 3e. Even in this case, it is possible to see a peak of colocalization superimposed to the random linear growing component. We subtracted the random distribution component as shown in Figure 3f, and we performed a Gaussian fit to extract an average distance value *d*_obj_ = 54 ± 32 nm (mean ± SD) which underestimates the expected distance *d* = 80 nm.

In summary, the object-based analysis allows extracting and mapping all the object-to-object distances within two-color images acquisitions. This is because the output of the object-based analysis consists of a list of all the coordinates between each particle of each channel, and of a map of each particles center of mass. With the coordinates data it is possible to define a colocalization nanometer distance and thanks to the map it is possible to know where in the sample these co-localization events occur. However, one of the drawbacks found in the object based-method, is that it was hard to define a correct threshold of the images because, even at the resolution provided by SIM, some nanorulers in the red channel were not fully resolved. To overcome this problem, we decided to raise up the threshold to separate as much as possible all the structures. Nevertheless, in some cases the plugin would find the center of mass in the middle between two red nanoruler features, thus significantly decreasing the resulting distance (Figure 3c).

### 3.3. SIM-ICCS Analysis of Optical Nanorulers

**Figure 4:**
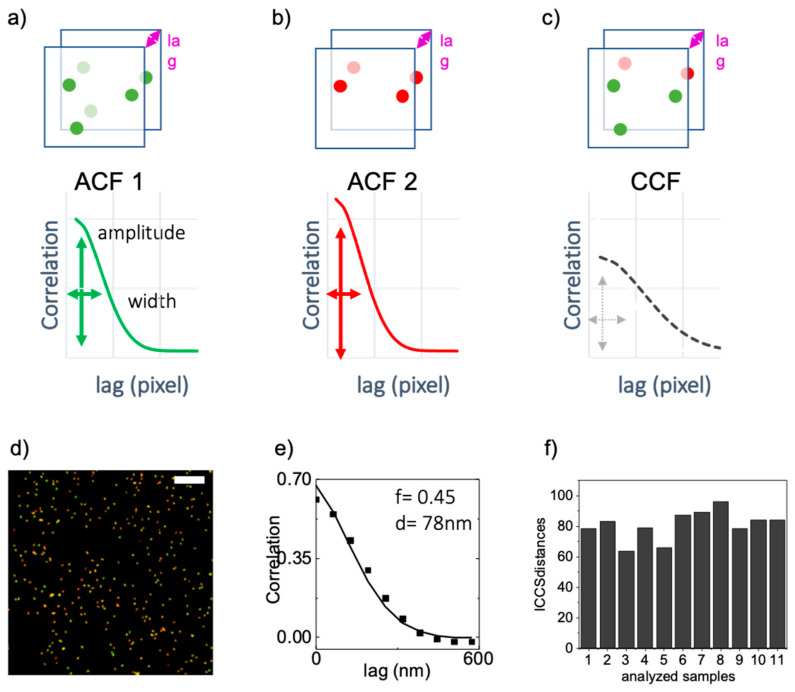
Image Cross-Correlation Spectroscopy (ICCS) analysis of optical nanorulers. (**a**–**c**) Schematic representation of the ICCS analysis. The amplitude and width of the Cross-Correlation Function (CCF) (**c**) are compared with the amplitude and width of the Auto Correlation Functions (ACF) of the single channel images (**a**,**b**) to extract a value of colocalization fraction and a value of correlation distance. (**d**,**e**) SIM reconstructed super-resolution image (512 × 512 pixel ROI) and corresponding image cross-correlation function. The colocalization fraction value (**f**) and average correlation distance (**d**) are indicated. Scalebar 5 µm. (**f**) Plot of ICCS distance values found in the 11 samples analyzed, from which we calculated the average distance d = 80 ± 9 nm (Mean ± SD).

In ICCS, the amplitude and width of the cross-correlation function (Figure 4c) are compared to the corresponding parameters of the autocorrelation functions (Figure 4a,b) to extract (i) the fraction of the cross-correlated particles in the sample and (ii) information about the average distance between correlated particles (Figure 4a–c) [29]. Indeed, in super-resolved ICCS, the resulting shape of the cross-correlation function strictly depends on the spatial resolution achieved by the microscope in both channels and on the distance, *d*, between the two labeled molecules. Considering the image of one red and one green molecule, at a relative distance *d*, the corresponding 2D auto correlation function will be characterized by a width w_11_ and w_22_. The 2D cross-correlation function, will be shifted from the origin of a distance *d*, and will have a width equal to w_cc_ = [(w_11_^2^ + w_22_^2^)/2)]^1/2^. If we now consider particles oriented in multiple direction relative to each other, the average cross-correlation function will show an increment in the width and a decreased amplitude value. The enlargement of the cross-correlation function width is estimated as Δw = w_12_-w_cc_ and this parameter Δw depends only on the average distance between the correlated particles [29].

We performed the SIM-ICCS analysis on the same *n* = 11 images of nanorulers. In the ICCS analysis, as expected, we obtained a positive cross-correlation function (Figure 4e). The mean value of the colocalized fraction extracted from all the images was *f*_ICCS_ = 1.11 ± 0.51 (mean ± SD). The mean distance value extracted by ICCS was *d*_ICCS_ = 80 ± 9 nm (mean ± SD), which agrees with the real distance *d* = 80 nm. The error is smaller compared to the object-based technique probably because ICCS is an average method and the values of the distances *d*, extracted by ICCS represent already average values for each image.

In ICCS, compared to the object-based method, the determination of distance was less affected by the non-optimal resolution of the SIM images. Indeed, ICCS is a statistical approach and does not require the target structures to be well-resolved and identified as objects thus making SIM-ICCS a good choice to study and analyze nanometer distances in any multicolor images, even in crowded acquisitions.

### 3.4. SIM-ICCS Analysis on Biological Samples

We finally tested the analysis on a model biological sample, the PR9-U937 cell line [32], by comparing the spatial distribution of two different pairs of functional nuclear sites. We performed both the object-based analysis and SIM-ICCS on a sample containing colocalizing nuclear sites and on a sample containing non-colocalizing nuclear sites.

**Figure 5:**
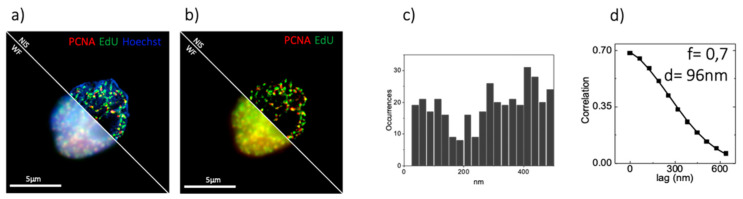
Biological sample of colocalizing nuclear markers. (**a**) Comparison between three color WF and SIM acquisition of PR9-U937 cell line. DNA is stained in blue by using Hoechst, Replication is stained by labeling EdU in green, Replication is marked by labeling RNApol2ser2P in red. (**b**) Two color considered for the colocalization analysis, EdU and RNApol2ser2P. (**c**) Object based analysis on the sample shown in (**b**). (**d**) ICCS analysis on the sample shown in (**b**).

Figure 5 shows a SIM image of DNA replication sites in a PR9-U937 cell, labeled with two different methods: by immunostaining of Proliferating Cell Nuclear Antigen (PCNA), a protein involved in the replication process acting as a processivity factor and by incorporation of a thymidine analog 5-ethynyl-2′-deoxyuridine (EdU). We analyzed the distribution of PCNA and EdU by the object-based RDD and by SIM-ICCS. The object-based RDD shows a colocalization peak superimposed to a random component. By subtracting the random component and by fitting the colocalization peak with a gaussian function, we find an average value of distance of d = 76 ± 41 nm (mean ± SD), as shown in Figure 5c. By SIM-ICCS we obtained, as expected, a positive CCF, corresponding to a colocalization fraction value of f = 0.7. The value of the mean distance, d, recovered by SIM-ICCS was d = 96 nm as shown in Figure 5d. Both the object-based and SIM-ICCS analysis confirmed the correlated distributions of PCNA and EdU, as they are both DNA replication foci markers.

**Figure 6:**
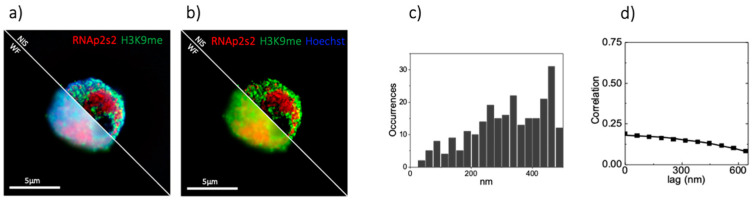
Biological sample of non-colocalizing nuclear markers**.** (**a**) Comparison between three color WF and SIM acquisition of PR9-U937 cell line. DNA is stained in blue by using Hoechst, gene silencing is stained by labeling H3K9me2 in green, active transcription is marked by labeling RNApol2ser2P in red. (**b**) Two color considered for the colocalization analysis, H3K9me2 and RNApol2ser2P. (**c**) Object based analysis on the sample shown in (**b**). (**d**) ICCS analysis performed on the sample shown in (**b**).

Figure 6 shows a SIM image of non-colocalizing nuclear structures in a PR9-U937 cell. We marked inactive chromatin by labeling histone H3K9me2, and transcriptionally active chromatin by labeling RNA polymerases 2 phosphorylated at the serine 2, RNApol2ser2P. As expected, in the object-based analysis we did not find any colocalization peak, indicating that the two nuclear markers are spatially uncorrelated, as shown in Figure 6c. By SIM-ICCS we find a CCF with a much lower amplitude (Figure 6d). Even if the amplitude is different from zero, application of the ICCS algorithm, as reported in Oneto et al. [29], yields a value of the colocalization fraction f = 0. This can be explained by the specific settings of the ICCS algorithm: we set f_ICCS_ = 0 when we have CCF fits with a χ^2^ value 50 times larger than the χ^2^ value of the ACF fit or when fits show a CCF width w_12_ too different from the average of the ACFs width (r_w_ < 0.5 or r_w_ > 2 with r_w_ = w_12_/(w_11_w_22_)^1/2^). These conditions are necessary to discard non-meaningful cross-correlation function. For instance, in Figure 5d, the non-zero cross-correlation function is probably due to an overlap of background features in the image. In this case, the algorithm does not calculate a correlation distance value.

## 4. Discussion

In this work, we showed that is possible to couple structured illumination microscopy and image cross-correlation spectroscopy in the co-localization analysis of two-color sample images. We validated our results by using a model sample and by comparing SIM-ICCS with an object-based analysis approach. In particular, we have analyzed the broadening of the cross-correlation function to provide quantitative information on the nanoscale distance between particles of different color, as demonstrated by Oneto et al. in the context of STED-ICCS [29]. The average correlation distance is an additional parameter that can be used in the quantitative analysis of 2-color SIM images by ICCS. The statistical error associated to the determination of distance was ~30 nm in the object-based analysis and ~10 nm in ICCS analysis. These values are in keeping with those reported by Oneto et al. [29]. The error in the object-based analysis depends on the ability to localize the center of the single objects in the images [29]. The error associated to the ICCS analysis is lower because each value of distance was calculated from an image containing several hundreds of identical nanorulers. We can say that, at the conditions of our experiments, the distance between two single objects can be measured with an error of ~30 nm (object-based analysis), whilst the mean value of distance can be measured with an error of ~10 nm (ICCS analysis on an image containing several hundreds of objects).

The first aspect that emerged from this work is that SIM-object-based analysis requires the single objects to be well resolved and isolated in order to assign them the x, y coordinates. When two structures of the same channel are not well resolved, the algorithm may find the center of mass inaccurately between the two. This will give an incorrect estimation of the distances between the two-color images if many structures are not well resolved. In contrast, ICCS analysis does not need a segmentation of the image, avoiding completely this problem. This suggests that ICCS analysis can be a valuable tool in high densities samples where object-based analysis might be less accurate. Moreover, with regards to samples with high-density of objects, the computational time required to perform the ICCS analysis will be more or less the same, based only on the number of pixels. On the contrary, the object-based analysis will be way slower because it depends on the number of objects detected in the image.

A second aspect is related to the fact that with ICCS analysis the correlation function only tells us if there is co-localization between the two targets, but it does not tell anything about where they co-localize. Instead, in the object-based method, the output of the analysis is a co-localization map showing all the ‘hotspots’ of co-localization and the corresponding values of object-object distance. In this respect, Oneto et al. [29] have shown that it is possible to produce ICCS maps from the data by performing a local analysis, namely by calculating, iteratively, the correlation function on smaller regions of the image [31,33]. Another possible strategy is to restrict the analysis on specific regions based on a selection mask. For instance, one can restrict the analysis to heterochromatic domains of the nucleus by selecting the regions of the image with a high signal of a DNA-binding dye [34,35].

## 5. Conclusions

In summary, we have shown that combination of structured illumination microscopy (SIM) with ICCS (SIM-ICCS) is a simple approach to quantify sub-diffraction distances from multi-color SIM images. The approach has been validated on SIM images of optical nanorulers and by a comparison with an object-based analysis performed on the same images. We believe that SIM-ICCS can be a valuable tool for quantifying the spatial distribution of molecules in cells.

## Data Availability

The data presented in this study are available on request from the corresponding author.
